# Accelerated Bone Regeneration by Adrenomedullin 2 Through Improving the Coupling of Osteogenesis and Angiogenesis via β-Catenin Signaling

**DOI:** 10.3389/fcell.2021.649277

**Published:** 2021-04-14

**Authors:** Feng Wang, Wenbo Wang, Lingchi Kong, Li Shi, Mengwei Wang, Yimin Chai, Jia Xu, Qinglin Kang

**Affiliations:** Department of Orthopaedic Surgery, Shanghai Jiao Tong University Affiliated Sixth People’s Hospital, Shanghai, China

**Keywords:** adrenomedullin 2, bone regeneration, bone marrow mesenchymal stem cell, angiogenesis, distraction osteogenesis

## Abstract

Both osteogenic differentiation and the pro-angiogenic potential of bone marrow mesenchymal stem cells (BMSCs) contribute to bone regeneration during distraction osteogenesis (DO). Adrenomedullin 2 (ADM2), an endogenous bioactive peptide belonging to the calcitonin gene-related peptide family, exhibits various biological activities associated with the inhibition of inflammation and the attenuation of ischemic-hypoxic injury. However, the effects and underlying mechanisms of ADM2 in osteogenic differentiation and the pro-angiogenic potential of BMSCs, along with bone regeneration, remain poorly understood. In the present study, we found that osteogenic induction enhanced the pro-angiogenic potential of BMSCs, and ADM2 treatment further improved the osteogenic differentiation and pro-angiogenic potential of BMSCs. Moreover, the accumulation and activation of β-catenin, which is mediated by the inhibition of nuclear factor kappa-light-chain-enhancer of activated B cells (NF-κB) and the activation of protein kinase B (AKT), have been shown to contribute to the effects of ADM2 on BMSCs. *In vivo*, ADM2 accelerated vessel expansion and bone regeneration, as revealed by improved radiological and histological manifestations and the biomechanical parameters in a rat DO model. Based on the present results, we concluded that ADM2 accelerates bone regeneration during DO by enhancing the osteogenic differentiation and pro-angiogenic potential of BMSCs, partly through the NF-κB/β-catenin and AKT/β-catenin pathways. Moreover, these findings imply that BMSC-mediated coupling of osteogenesis and angiogenesis may be a promising therapeutic strategy for DO patients.

## Introduction

Distraction osteogenesis (DO), which exhibits unique treatment advantages for bone defect repair and osseous deformity correction, is widely employed worldwide in the field of trauma and orthopedic surgery (Mauffrey et al., 2015; Quinnan, 2017; Aktuglu et al., 2019). Gradual rhythmic traction was applied using an external fixator in the DO process to fully induce neo-osteogenesis within the distraction zone between the proximal and distal bone segments (Ilizarov, 1988; Kong et al., 2020). Despite the novel osteogenesis-inducing ability, the DO technique requires a lengthy consolidation phase, during which the unwieldy external fixator will inevitably increase patient discomfort and the associated complications (Spiegl et al., 2013). Hence, accelerating the callus consolidation and shortening the treatment duration are of great clinical significance.

The skeleton is a highly vascularized tissue containing an abundant capillary network, which not only provides access to nutrients and oxygen, but also secretes various cytokines, regulating the local osteogenic microenvironment (Stegen et al., 2015; Ramasamy et al., 2016; Hendriks and Ramasamy, 2020; Stucker et al., 2020). Kusumbe defined type-H endothelial cells, which exhibit important functions in the regulation of osteogenesis and angiogenesis, based on the criterion of a high expression of platelet endothelial cell adhesion molecule-1 and endomucin (CD31^hi^EMCN^hi^) (Kusumbe et al., 2014). Hence, the theoretical system of the coupling between osteogenesis and angiogenesis in the skeletal system was gradually established and perfected (Ramasamy et al., 2014; Langen et al., 2017; Xu et al., 2019). In recent years, with the accumulation of knowledge about the coupling of osteogenesis and angiogenesis, the pivotal role of bone marrow mesenchymal stem cells (BMSCs) in bone regeneration has been further explored. On one hand, both the substance supply and signaling regulation functions of the intraosseous capillary system are largely dedicated to improve the recruitment and osteogenic differentiation of BMSCs (Kusumbe et al., 2014). On the other hand, osteoblasts differentiated from BMSCs are also characterized as important regulatory cells for type-H endothelial cells and angiogenesis (Xu et al., 2018). Therefore, the simultaneous promotion of osteogenic differentiation and the pro-angiogenic potential of BMSCs may represent an ideal therapeutic strategy for accelerating bone regeneration during DO, based on the coupling of osteogenesis and angiogenesis.

Adrenomedullin 2 (ADM2), also known as intermedin, is an endogenous peptide belonging to the calcitonin gene-related peptide (CGRP)/calcitonin family (Naot et al., 2019). Studies have shown that ADM2 is ubiquitously expressed in various tissues and has diverse biological activities, including the induction of endothelial cell proliferation (Wang et al., 2018), amelioration of ischemia/reperfusion injury (Song et al., 2009; Qiao et al., 2013), and inhibition of the inflammatory response and oxidative stress (Lu et al., 2016; Xiao et al., 2018). Meanwhile, most peptides in the calcitonin family possess similar biological activities, and the pharmacological effects of CGRP and ADM, which share the dimers of calcitonin receptor-like receptor (CLR) and receptor-modifying proteins (RAMPs) as common receptors, especially CLR/RAMP1 and CLR/RAMP3, with ADM2, are the most similar to those of ADM2 (Naot et al., 2019). Therefore, previous investigations of CGRP and ADM have been of great significance for studying ADM2 in the areas of osteogenesis, angiogenesis, and bone regeneration (Cornish et al., 2001; Zhou et al., 2016; Mi et al., 2021), in which the biological functions of ADM2 have rarely been investigated. Of note, the most reported biological functions of ADM2 are mediated through the intracellular activation of protein kinase B (AKT) and the inhibition of nuclear factor kappa-light-chain-enhancer of activated B cells (NF-κB) (Song et al., 2009; Teng et al., 2011; Li et al., 2013). Based on previous studies, NF-κB signaling promotes β-catenin ubiquitination and degradation through the induction of the Smad ubiquitin regulatory factor (Smurf) 1 and Smurf2 (Chang et al., 2013), and AKT signaling activates β-catenin through the phosphorylation and inactivation of glycogen synthase kinase-3β (GSK-3β) (Zheng et al., 2018). Hence, NF-κB and AKT may synergistically regulate the expression and activation of β-catenin, which has been widely reported to play a vital role in improving osteogenesis and angiogenesis (Houschyar et al., 2018; Martowicz et al., 2019; Zhang et al., 2020). Therefore, ADM2 might have a stimulatory effect on bone regeneration by improving the osteogenic differentiation and the pro-angiogenic potential of BMSCs through the accumulation and activation of β-catenin.

In the present study, we investigated whether ADM2 could improve the osteogenic differentiation and pro-angiogenic potential of BMSCs. Additionally, the role of β-catenin, which is regulated by NF-κB and AKT signaling, on the effects of ADM2 on BMSCs was also explored. Furthermore, a rat DO model was employed to examine the *in vivo* effects of ADM2 on bone regeneration and the coupling of osteogenesis and angiogenesis.

## Materials and Methods

### Cell Culture

Bone marrow mesenchymal stem cells were freshly harvested from the bone marrow of 2-week-old Sprague-Dawley (SD) male rats by flushing the femur and tibia. Flushed bone marrow cells were cultured in modified Eagle’s medium alpha (HyClone, United States) supplemented with 10% fetal bovine serum (FBS; Gibco, United States) and 1% penicillin-streptomycin (P/S; Gibco). Upon 80 – 90% confluence, the cells were treated with trypsin and re-plated for expansion. After characterization by differentiating the cells into cells of the three lineages and using a flow cytometry, as previously described (Yang et al., 2018), the BMSCs between passages two and four were used in the downstream experiments. The endothelial Ea.hy926 cells were cultured in high-glucose Dulbecco’s modified Eagle’s medium (HyClone) containing 10% FBS and 1% P/S. Cells were cultured at 37°C in a humidified atmosphere containing 5% CO_2_.

### Osteogenic Differentiation

To determine the effects of ADM2 on the osteogenic differentiation of BMSCs, the alkaline phosphatase (ALP) activity and mineral deposition were measured. Briefly, BMSCs were seeded in 24-well plates (5 × 10^4^/well). At 80% confluence, the medium was replaced with osteogenic induction medium (OIM, 20 mM β-glycerophosphate, 1 nM dexamethasone, and 50 μM L-ascorbic acid-2-phosphate in complete medium; Sigma-Aldrich, United States) containing serial concentrations of ADM2 (1 – 2,000 nm; Phoenix Pharmaceuticals, United States), and the medium was refreshed every 2 days. MK2206 (100 nM; MedChemExpress, United States) and tumor necrosis factor-alpha (TNF-α, 10 ng/mL; PeproTech, United States) were used to verify the molecular mechanism by which ADM2 regulates the osteogenic differentiation of BMSCs. ALP staining and activity assays were performed 7 days after osteogenic induction, according to the manufacturer’s instructions (Beyotime, China). On the 14th day of differentiation, alizarin red S (ARS; Cyagen Biosciences, China) staining was performed to evaluate the mineral deposition. For quantitative analysis of mineralization, calcium deposition was eluted using 10% (v/v) cetylpyridinium chloride (Sigma-Aldrich), and the OD value was measured at 570 nm.

### Quantitative Real-Time PCR (qRT-PCR) Analysis

After inducing differentiation for 7 days in 6-well plates, the total cellular RNA was extracted using an RNA Purification Kit (EZBioscience, United States), and cDNA was obtained from 500 ng of total RNA using a Reverse Transcription Kit (EZBioscience). Next, qRT-PCR was performed using SYBR Green qPCR Master Mix (EZBioscience). The relative gene expression was calculated by the 2^–ΔΔCT^ method, and *GAPDH* was used as a reference for normalization. The primers were purchased from BioTNT (China) and primer sequences are shown in Table 1.

**TABLE 1:** Primer sequences.

**Gene**	**Forward (5′–3′)**	**Reverse (5′–3′)**
*ALP*	CCGCAGGATGTGAACTACT	GGTACTGACGGAAGAAGGG
*Ang-2*	GAAGAAGGAGATGGTGGAGAT	CGTCTGGTTGAGCAAACTG
*Ang-4*	GCTCCTCAGGGCACCAAGTTC	CACAGGCGTCAAACCACCAC
*CLR*	CAACAGCACGCATGAGAAAGTG	GTAATCCGTTGGCAACTTAGGC
*OCN*	CAGACAAGTCCCACACAGCA	CCAGCAGAGTGAGCAGAGAGA
*OPN*	GGCCGAGGTGATAGCTT	CTCTTCATGCGGGAGGT
*OSX*	GGAAAAGGAGGCACAAAGAA	CAGGGGAGAGGAGTCCATT
*RAMP1*	CATCCAGGAGCTGTGTCTCA	AATGGGGAGCACAATGAAAG
*RAMP3*	GTATGCGGTTGCAATGAGACA	TCTTCTAGCTTGCCAGGCAC
*RUNX2*	ACTTCCTGTGCTCGGTGCT	GACGGTTATGGTCAAGGTGAA
*SDF-1*	GCGTCTATGTCTTGTTTGGAA	TACCTCATACACAGCCTTTGC
*Slit3*	GCTAAACCAGACCCTGAACCT	ACTGTTGATGCCCACTGCT
*VEGFA*	CACGACAGAAGGGGAGCAGAAAG	GGCACACAGGACGGCTTGAAG
*GAPDH*	ATGGCTACAGCAACAGGGT	TTATGGGGTCTGGGATGG

### Western Blot Analysis

After induction of differentiation for 7 days in 6-well plates, the total cellular protein was obtained using RIPA lysis buffer, containing protease and protein phosphatase inhibitors (Solarbio, China), on ice. Protein concentration was determined using a BCA protein assay kit (EpiZyme, China). Proteins (30 μg) were subjected to 10% SDS-PAGE, transferred to a polyvinylidene difluoride membrane (Millipore, United States), and incubated overnight at 4°C with the primary antibodies after blocking with 5% bovine serum albumin (BSA). The primary antibodies used in this study included anti-AKT (1:1,500; #4691, Cell Signaling Technology, United States), anti-phosphorylated AKT (p-AKT; 1:1,000; #4060, Cell Signaling Technology), anti-p65 (1:1,500; ab16502, Abcam, United Kingdom), anti-phosphorylated p65 (p-p65; 1:1,000; ab76302, Abcam), anti-β-catenin (1:1,500; #8480, Cell Signaling Technology), anti-phosphorylated β-catenin (p-β-catenin; 1:1,000; #4176, Cell Signaling Technology), and anti-GAPDH (1:2,000; #5174, Cell Signaling Technology). Membranes were then incubated with horseradish peroxidase (HRP)-conjugated secondary antibody (1:10,000; 111-035-003, Jackson ImmunoResearch) at room temperature. The bands were visualized using enhanced chemiluminescence reagent (Millipore), and the grayscale of protein bands was semi-quantified (*n* = 3) using ImageJ software (National Institutes of Health, United States).

### Preparation of Conditioned Medium (CM)

After 7 days of osteogenic differentiation, the medium was replaced with complete medium for an additional 2 days of incubation. The CM from each group was harvested and centrifuged at 2,000 × *g* for 10 min to collect the supernatant, which was stored at –80°C for further experiments.

### Enzyme-Linked Immunosorbent Assay (ELISA)

VEGFA, SDF-1, and Slit3 concentration analyses of CM were performed using commercial ELISA kits (E04757r, E08729r, EL021769RA, CUSABIO, China). All procedures were conducted according to the manufacturer’s instructions, and the protein concentration values were calculated according to the standard curve.

### Angiogenesis-Related Assays *in vitro*

To test whether ADM2 could augment the pro-angiogenic ability of OIM-induced BMSCs, the endothelial Ea.hy926 cells were treated with CM from each group.

For the tube formation assay, Ea.hy926 cells (2 × 10^4^/well) were seeded onto 96-well plates coated with Matrigel (BD Biosciences, United States) and incubated in blank medium under different treatment conditions (supplemented with 20% CM from each group) for 6 h. The total tube length and total branch points were measured to evaluate the ability of tube formation.

For the Transwell migration assay, Ea.hy926 cells (2 × 10^4^/well) were loaded onto the top chamber of 24-well, 8 μm pore-size Transwell plates (Corning, United States), and then incubated with the CM from each group in the lower chamber for 6 h. Subsequently, unmigrated cells that remained in the upper chambers were removed using cotton swabs, while the migrated cells that passed through the membrane pores were fixed with 4% PFA for 15 min, stained with 0.5% crystal violet for 5 min, and counted using an optical microscope (Leica).

For the scratch wound assay, Ea.hy926 cells were seeded onto 6-well plates and cultured until they reached confluence. Next, the confluent layers of cells were scratched using a sterile pipette tip. After washing, the cells were incubated in blank medium under different treatment conditions (supplemented with 20% CM from each group). Images of the wounds were acquired immediately, 6 and 12 h later, the wound areas were measured using ImageJ software, and the migration area (%) was calculated.

### Animal Surgery and Treatment

All experimental procedures were approved by the Animal Research Committee of Shanghai Jiao Tong University Affiliated Sixth People’s Hospital (DWLL2021-0414). A total of 36 adult male Sprague-Dawley (SD) rats (350 – 400 g) were used in this study and randomly assigned to the control (*n* = 18) and ADM2 (*n* = 18) groups. To establish the DO model, a transverse osteotomy was performed at the midshaft of the right tibia after anesthesia and exposure. Then, a specially designed monoliteral external fixator (Tianjin Xinzhong Company, China) was mounted to fix the proximal and distal segments of the tibia. Thereafter, the surgical incisions were closed layer-wise. The periosteum was preserved as much as possible during the procedure. The DO treatment was comprised of three phases: latency phase for 5 days, distraction phase for 10 days (0.25 mm every 12 h), and consolidation phase for 4 weeks. ADM2 (200 μg/kg/day) and an equal volume of PBS were subcutaneously injected during the consolidation phase in each group.

### Digital Radiography and Micro-Computed Tomography (CT)

From the beginning of the consolidation phase, X-ray films focused on the distraction gap were acquired weekly. The lengthened tibia specimens were harvested 2 and 4 weeks after the distraction. Micro-CT scanning was performed to quantitatively evaluate bone regeneration within the distraction zone (*n* = 6 at each time point). Thereafter, the three-dimensional (3D) reconstruction of the lengthened tibia was performed using CTVox software (Bruker, Germany). Parameters, including the bone volume/tissue volume (BV/TV) and bone mineral density (BMD) of the regenerated bone, were analyzed using CTAn software (Bruker).

### Angiography

To evaluate the neovascularization of the distraction areas, cardiac Microfil (Flow Tech, United States) perfusion of rats was performed under general anesthesia after 2 weeks of consolidation (*n* = 3). The perfused rats were immediately sacrificed via cervical dislocation, and then stored at 4°C for 24 h to ensure the polymerization of the contrast agent. Thereafter, the tibia samples were harvested, fixed using 4% PFA at 4°C for 24 h, decalcified in 10% ethylenediamine tetraacetic acid (EDTA, pH 7.4), and subjected to micro-CT analyses with an isotropic resolution of 9 μm, followed by 3D reconstruction and analysis of the blood vessel volume using the CTVox and CTAn software packages.

### Biomechanical Testing

The mechanical characteristics of the fresh tibia specimens (*n* = 6) were determined using a four-point bending device (Hounsfield Test Equipment, United Kingdom) 4 weeks after distraction. During the test, the tibiae were loaded in the anterior-posterior direction with the posterior side under tension. The modulus of elasticity (E-modulus), ultimate load, and energy to failure were recorded and analyzed using Vernier Graphical Analysis software (Vernier, United States).

### Histological and Immunohistochemical Analyses

For histological analyses, after 2 (*n* = 3 per group) and 4 (*n* = 3 per group) weeks of consolidation, the tibia specimens were harvested, fixed in 4% PFA for 24 h, decalcified in 10% EDTA for 21 days, dehydrated using a graded ethanol series, and then embedded in paraffin. Samples were cut into 5-μm-thick longitudinally oriented sections and processed for hematoxylin-eosin (H&E), Masson’s trichrome, and Safranine O-Fast Green (SO-FG) staining.

For immunohistochemical staining, the sections were incubated in 0.3% hydrogen peroxide for 20 min to quench the endogenous peroxidase activity. After antigen retrieval in 0.01 mol/L citrate buffer (pH 6.0) at 95°C for 20 min and blocking with 5% goat serum for 1 h, sections were incubated with the primary antibodies at 4°C overnight. The sections were then incubated with the secondary antibodies (1:1,000; 111-035-003, Jackson ImmunoResearch) for 1 h at 37°C, counterstained with hematoxylin, and visualized using an HRP-streptavidin system. The primary antibodies used in this study included anti-OCN (1:100; A6205, ABclonal, China) and anti-β-catenin (1:100; #8480, Cell Signaling Technology) antibodies.

### Immunofluorescence Staining

To test the activation and nuclear translocation of β-catenin, BMSCs were fixed in 4% paraformaldehyde (PFA) and permeabilized using 0.1% Triton X-100 in phosphate-buffered saline (PBS) containing 5% BSA. After blocking with 5% BSA for 1 h, the cells were incubated with anti-β-catenin antibody (1:100; #8480, Cell Signaling Technology) overnight at 4°C. Subsequently, the cells were washed three times with PBS and incubated with Cy3-conjugated secondary antibody (1:1000; ab6939, Abcam) for 1 h, followed by incubation with 4’,6-diamidino-2-phenylindole (DAPI) for 15 min. The fluorescence signal was captured using a fluorescence microscope (DMi8, Leica, Germany).

CD31 and EMCN double immunofluorescence staining was performed to evaluate the extent of type-H vessel formation. After 2 weeks of consolidation, tibia specimens (*n* = 3) were harvested and decalcified in 18% EDTA (pH 7.4) for 7 days after fixation. Subsequently, the samples were dehydrated in 30% sucrose, embedded in optimal cutting temperature compound, and cut into 10-μm-thick longitudinally oriented sections. Bone sections were then incubated with the primary antibodies overnight at 4°C, followed by incubation with fluorophore-conjugated secondary antibodies at room temperature for 1 h. The nuclei were stained with DAPI. A fluorescence microscope (DMi8, Leica, Germany) was used for observation and image capture. The EMCN antibody was purchased from Santa Cruz Biotechnology (1:100; sc-65495, United States). CD31 antibody (1:100; ab64543) and all the secondary antibodies (1:200; ab97035, ab6840) were obtained from Abcam.

### Statistical Analysis

All data are presented as mean ± standard deviation. The statistical differences were analyzed using Student’s *t*-test or Mann–Whitney *U*-test between two *in vivo* groups and one-way analysis of variance (ANOVA), followed by Tukey’s *post hoc* test, among multiple *in vitro* groups, after Shapiro–Wilk normality test, using GraphPad Prism 8 software (GraphPad Software, United States). A two-tailed *P*-value of less than 0.05 was considered statistically significant.

## Results

### ADM2 Promoted the Osteogenic Differentiation of BMSCs

**FIGURE 1:**
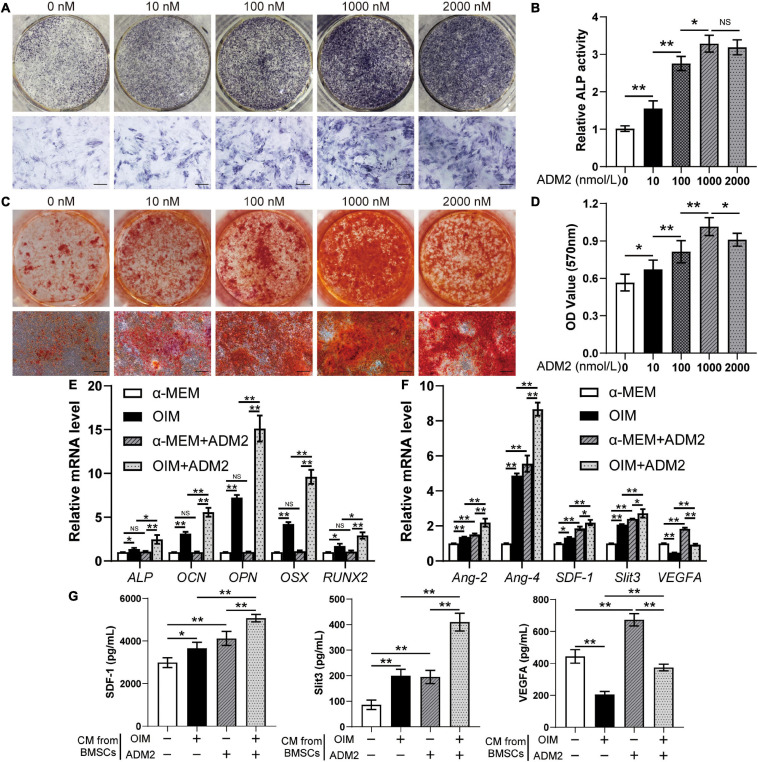
ADM2 promotes osteogenic differentiation and angiogenic factors expression of BMSCs. **(A–D)** Osteogenic differentiation of BMSCs treated with OIM and different concentrations of ADM2 were determined with ALP staining **(A)**, ALP activity assays **(B)**, and alizarin red staining **(C)**. Calcium deposition was assessed by measuring the optical density **(D)**. Scale bar: 200 μm. **(E,F)** Expression of osteogenic-specific genes **(E)** and angiogenic-specific genes **(F)** of BMSCs treated with complete medium, OIM, ADM2, and OIM + ADM2 (1000 nM) were assessed with qRT-PCR. **(G)** Detection of SDF-1, Slit3, and VEGFA concentrations in conditioned medium from BMSCs in each group using ELISA. The data were confirmed by one-way analysis of variance (ANOVA) followed by Tukey’s *post hoc* test from three independently repeated tests and are presented as the means ± SD. ^NS^*P* > 0.05, *P < 0.05, **P < 0.01.

After the three lineages of differentiation test (Supplementary Figure 1A) and flow cytometry (Supplementary Figure 1B), the characterized cell population of BMSCs was utilized for subsequent experiments. To evaluate the effect of ADM2 on the osteogenic differentiation of BMSCs, we stained and measured the activity of ALP, an early osteogenic differentiation marker, on the 7th day of induction. ADM2 (1 – 1,000 nM) significantly increased the activity of ALP in a dose-dependent manner, whereas BMSCs treated with a higher concentration of ADM2 (2,000 nM) failed to exhibit an enhanced ALP activity compared to those treated with 1000 nM ADM2 (Figures 1A,B). The results of ARS staining and calcium quantitative analysis subsequently confirmed that 1,000 nM ADM2 induced maximal mineralization (Figures 1C,D). Moreover, the gene expression of *CLR* and *RAMP1* was also elevated by ADM2 treatment in a dose-dependent manner up to 1,000 nM, although the expression of *RAMP3* was not detectable in BMSCs (Supplementary Figure 2). Consequently, the optimal concentration of ADM2 was determined to be 1,000 nM for further examination of the gene expression of osteogenic markers. As revealed by the results of qRT-PCR, the expression levels of osteogenic genes, including *ALP*, *OSX*, *OCN*, *OPN*, and *RUNX2*, in BMSCs were not upregulated by ADM2 treatment alone without osteogenic induction, while those in OIM-induced BMSCs were significantly elevated by ADM2 supplementation, suggesting the promotive effect of ADM2 on the osteogenic differentiation of BMSCs (Figure 1E).

### ADM2 Enhanced the Pro-angiogenic Potential of OIM-Induced BMSCs

Primarily, the gene expression of several angiogenic factors in BMSCs was examined after osteogenic differentiation and ADM2 treatment to evaluate their pro-angiogenic potential. With ADM2 treatment alone, there were various degrees of upregulation of the expression levels of all the angiogenic genes examined (Figure 1F). Meanwhile, a higher expression of *Ang-2*, *Ang-4*, *SDF-1*, and *Slit3*, a novel angiogenic factor associated with type-H vessels, was observed in the OIM group, and the expression of these genes was further elevated by ADM2 treatment (Figure 1F). However, *VEGFA* gene expression decreased during the osteogenic induction of OIM (Figure 1F). Interestingly, ADM2 treatment partially rescued *VEGFA* gene expression, even though it enhanced the osteogenic differentiation of BMSCs (Figure 1F). The ELISA results subsequently confirmed the enhanced pro-angiogenic potential of ADM2-treated BMSCs, with or without osteogenic differentiation, as revealed by elevated concentrations of multiple angiogenic factors, including SDF-1, Slit3, and VEGFA, in the CM from the α-MEM + ADM2 and OIM + ADM2 groups compared with the α-MEM and OIM groups, respectively (Figure 1G). Meanwhile, the upregulation of *SDF-1* and *Slit3* and the downregulation of *VEGFA* in BMSCs during osteogenic differentiation were also confirmed in the CM via cytokine detection using ELISA.

**FIGURE 2:**
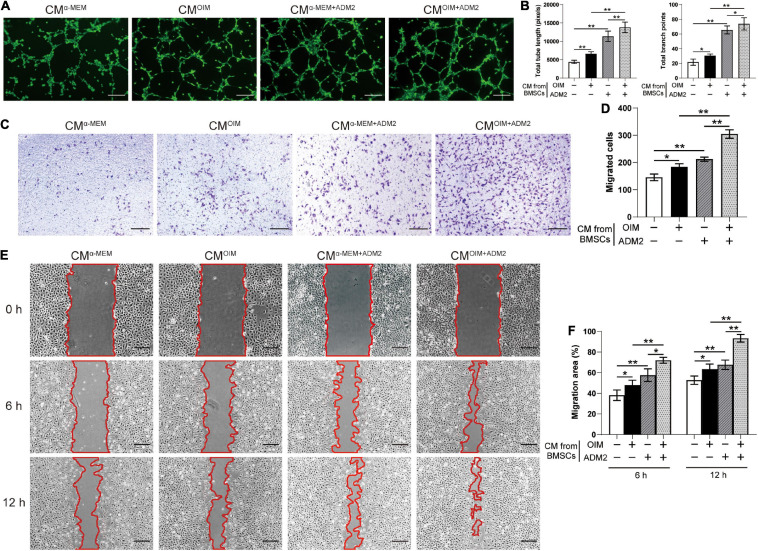
Osteogenic induction and ADM2 treatment gradually promote the pro-angiogenic ability of BMSCs. **(A,B)** Representative images **(A)** and quantification of tube formation **(B)** in endothelial cells stimulated with conditioned medium from BMSCs treated with complete medium, OIM, ADM2, and OIM + ADM2 (1000 nM). Scale bar: 200 μm. **(C–F)** Endothelial cells motility in each group was evaluated using the Transwell migration assay **(C,D)** and the scratch wound assay **(E,F)**. Scale bar: 200 μm. The data were confirmed by one-way analysis of variance (ANOVA) followed by Tukey’s *post hoc* test from three independently repeated tests and are presented as the means ± SD. *P < 0.05, **P < 0.01.

To further elucidate the effects of the CM from BMSCs in each group on angiogenesis, the endothelial Ea.hy926 cells were employed for a series of angiogenesis-related assays *in vitro*. Endothelial cells treated with CM^OIM^ and CM^α^ ^–MEM+ADM2^ formed a higher number of capillary tube-like structures, with increased total tube length and number of branch points, compared to those treated with CM^α^ ^–MEM^, and the combination of both OIM induction and ADM2 treatment exhibited a synergistic stimulative effect on the pro-angiogenic potential of CM, forming optimal capillary tube-like structures (Figures 2A,B). As evidenced by the Transwell assay results, CM^OIM^ and CM^α^ ^–MEM+ADM2^ induced more endothelial cells to migrate to the lower side of the membrane, and the CM from the OIM + ADM2 group resulted in the greatest increase in endothelial cell migration among all the groups (Figures 2C,D). Moreover, the scratch wound assay revealed a similar tendency that the CM from OIM-induced or ADM2-treated BMSCs both induced a higher endothelial cell migration compared to the α-MEM group (Figures 2E,F). Furthermore, CM^OIM+ADM2^ induced an optimal speed of scratch wound healing, resulting in a nearly continuous cell layer across the wound after 12 h of migration (Figures 2E,F), suggesting the enhanced pro-angiogenic potential of BMSCs induced by OIM induction and ADM2 treatment.

### ADM2 Promoted the Accumulation and Activation of β-Catenin Signaling in BMSCs

**FIGURE 3:**
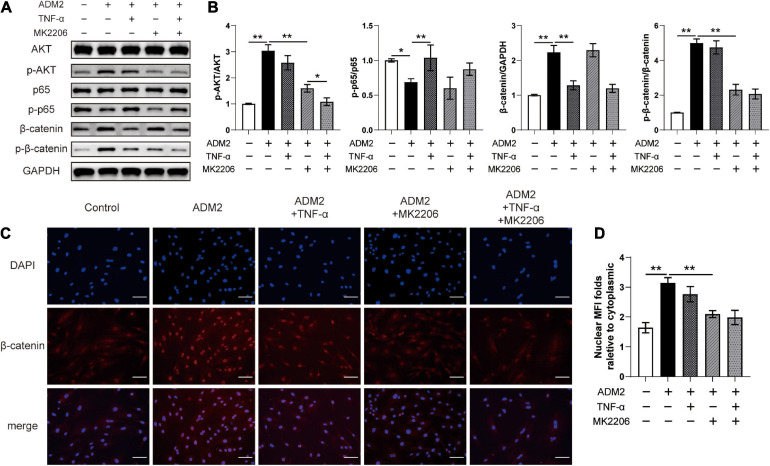
ADM2 regulates the NF-κB/β-catenin and AKT/β-catenin signaling in BMSCs. **(A)** Western blot of AKT, p-AKT, p65, p-p65, β-catenin, and p-β-catenin in BMSCs treated with OIM, OIM + ADM2, OIM + ADM2 + TNF-α, OIM + ADM2 + MK2206, and OIM + ADM2 + TNF-α + MK2206. **(B)** Quantitative analysis in the expression of β-catenin and phosphorylated levels of AKT, p65, and β-catenin. **(C)** Representative immunocytochemistry images showing the expression and distribution of β-catenin in BMSCs from each group. Scale bar: 100 μm. **(D)** Quantitative analysis of the nuclear translocation of β-catenin. The data were confirmed by one-way analysis of variance (ANOVA) followed by Tukey’s *post hoc* test from three independently repeated tests and are presented as the means ± SD. ^∗^*P* < 0.05, ^∗∗^*P* < 0.01. MFI, mean fluorescence intensity.

As revealed by the results of western blotting, ADM2 inhibited p65 and activated AKT in OIM-induced BMSCs, subsequently upregulating the expression and phosphorylation levels of β-catenin. However, TNF-α and MK2206, a specific inhibitor of AKT, partially attenuated ADM2-induced accumulation and the activation of β-catenin, respectively (Figures 3A,B). Moreover, the expression of phosphorylated β-catenin was mostly attenuated upon treatment with MK2206 and TNF-α in ADM2-induced BMSCs (Figures 3A,B). These significant variations in the expression and activation state of β-catenin induced by ADM2, TNF-α, and MK2206 were further confirmed using immunofluorescence analysis. The results showed an elevated nuclear mean fluorescence intensity (MFI) relative to cytoplasm, which presented an optimal activation and nuclear translocation of β-catenin, in the ADM2 group and attenuated MFI folds in the other experimental groups (Figures 3C,D). Of note, TNF-α activated the NF-κB pathway, which promoted the degradation of β-catenin, and ultimately reduced the expression levels of both total β-catenin and phosphorylated β-catenin (Figures 3A,B). However, MK2206 inactivated AKT signaling and slightly reduced the phosphorylation of p65, leading to a reduction in the phosphorylation level of β-catenin, while having little effect on the expression of total β-catenin (Figures 3A,B). Moreover, when both interventions were applied simultaneously, the expression of total β-catenin was similar to that observed upon TNF-α treatment alone, while the phosphorylation level of β-catenin was significantly lower than that observed via separate TNF-α or MK2206 application (Figures 3A,B).

**FIGURE 4:**
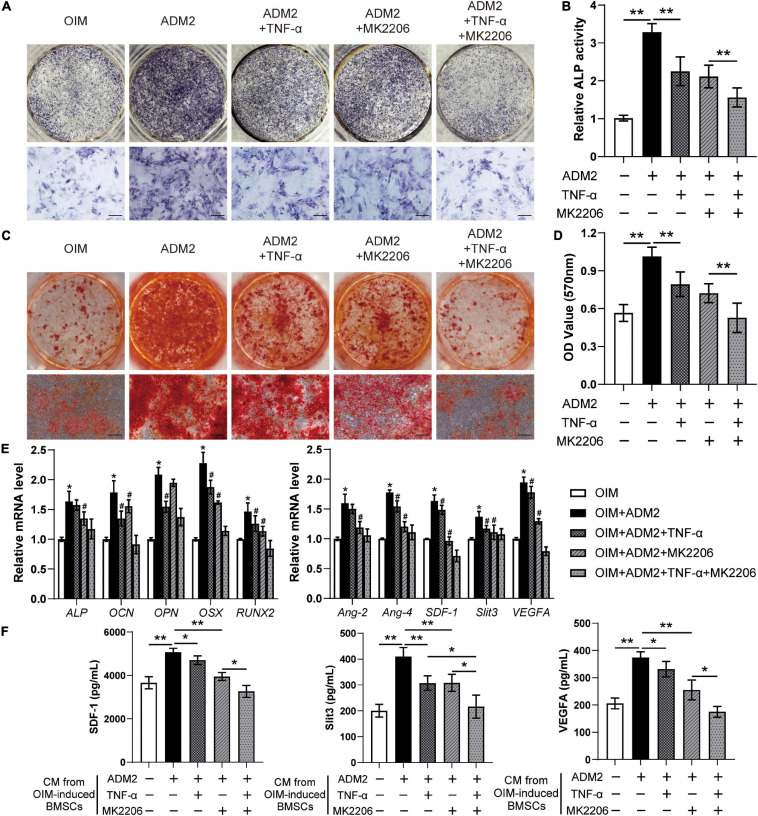
TNF-α and MK2206 attenuate ADM2-improved osteogenic differentiation and angiogenic factors expression of BMSCs. **(A–D)** Osteogenic differentiation of BMSCs treated with OIM, OIM + ADM2, OIM + ADM2 + TNF-α, OIM + ADM2 + MK2206, and OIM + ADM2 + TNF-α + MK2206 were determined with ALP staining **(A)**, ALP activity assays **(B)**, and alizarin red staining **(C)**. Calcium deposition was assessed by measuring the optical density **(D)**. Scale bar: 200 μm. **(E)** Expression of osteogenic-specific genes and angiogenic-specific genes of BMSCs in each group were assessed using qRT-PCR. **(F)** Detection of SDF-1, Slit3, and VEGFA concentrations in conditioned medium from BMSCs in each group using ELISA. The data were confirmed by one-way analysis of variance (ANOVA) followed by Tukey’s *post hoc* test from three independently repeated tests and are presented as the means ± SD.*P < 0.05, **P < 0.01, ^#^*P* < 0.05 vs. OIM + ADM2 group.

**FIGURE 5:**
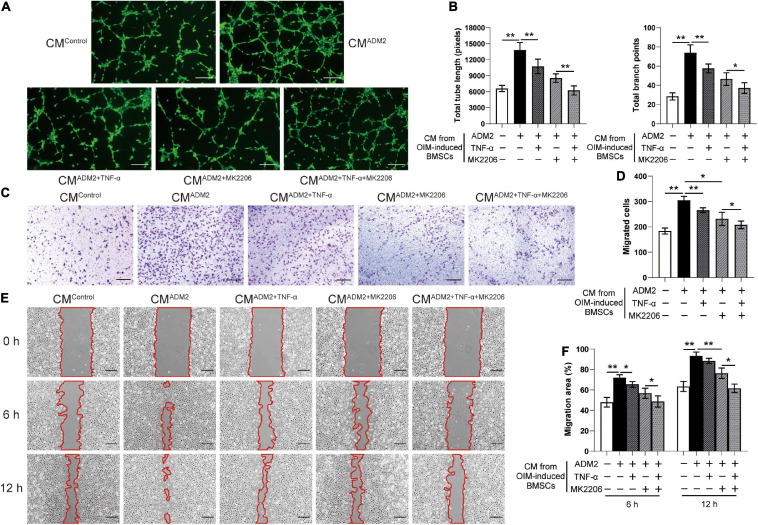
TNF-α and MK2206 attenuate ADM2-improved pro-angiogenic ability of BMSCs. **(A,B)** Representative images **(A)** and quantification of tube formation **(B)** in endothelial cells stimulated with conditioned medium from BMSCs treated with OIM, OIM + ADM2, OIM + ADM2 + TNF-α, OIM + ADM2 + MK2206, and OIM + ADM2 + TNF-α + MK2206. Scale bar: 200 μm. **(C–F)** Endothelial cell motility in each group was evaluated using the Transwell migration assay **(C,D)** and the scratch wound assay **(E,F)**. Scale bar: 200 μm. The data were confirmed by one-way analysis of variance (ANOVA) followed by Tukey’s *post hoc* test from three independently repeated tests and are presented as the means ± SD. *P < 0.05, **P < 0.01.

The effects of TNF-α and MK2206 on the osteogenic differentiation and pro-angiogenic potential of ADM2-induced BMSCs were investigated to further verify the mechanism by which ADM2 activates BMSCs. As revealed by the results of ALP activity and ARS analysis, either TNF-α or MK2206 attenuated the ADM2-improved osteogenic differentiation of BMSCs, and the combination of both interventions exhibited a synergistic inhibitory effect (Figures 4A–D). The detection of the expression of osteogenic genes further confirmed the adverse effects of TNF-α and MK2206 on the osteogenic differentiation of ADM2-treated BMSCs (Figure 4E). Additionally, the results of qRT-PCR and ELISA demonstrated that the gene and protein expression of multiple angiogenic factors was also reduced at different levels by the administration of TNF-α and MK2006 independently, or by simultaneous administration (Figures 4E,F), indicating the impaired angiogenic potential of BMSCs induced by OIM and ADM2. Subsequently, endothelial cells were used to confirm the angiogenic activity of the CM from each group. Endothelial cells treated with CM^ADM2^ formed the highest number of capillary tube-like structures on Matrigel among all the groups; once BMSCs were induced with TNF-α and MK2206, the pro-angiogenic effect of their CM was attenuated (Figures 5A,B). Meanwhile, the results of the Transwell assay and scratch wound assay revealed similar results regarding endothelial cell motility, with an optimal number (Figures 5C,D) and area (Figures 5E,F) of migrated cells in the CM^ADM2^ group and a differently impaired mobilization effect of the CM from ADM2-treated BMSCs upon TNF-α and MK2206 treatment.

### ADM2 Accelerated Bone Consolidation During Distraction Osteogenesis

**FIGURE 6:**
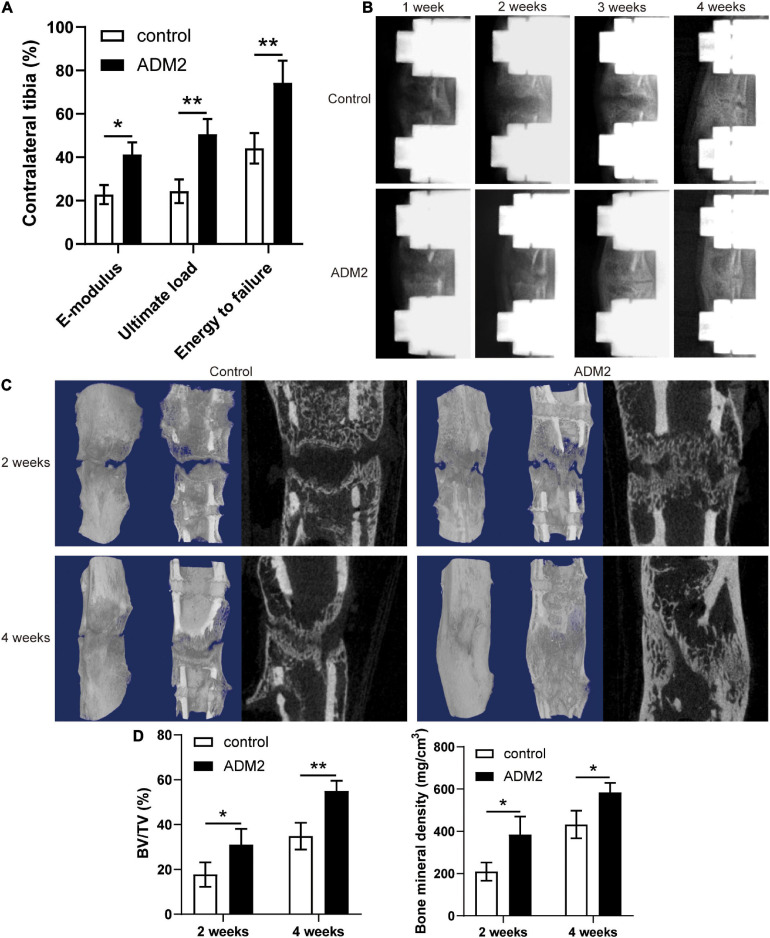
ADM2 administration accelerates bone consolidation during distraction osteogenesis in rats. **(A)** Mechanical properties, including E-modulus, ultimate load, and energy to failure of distraction regenerates in control (*n* = 6) and ADM2 (*n* = 6) groups. The values were normalized to the corresponding contralateral normal tibias. **(B)** Representative X-rays of distraction regenerates at various time points. **(C,D)** Representative 3D and longitudinal images **(C)** and quantitative analysis **(D)** of micro-CT data, including bone mineral density (BMD) and bone volume/tissue volume (BV/TV), of the tibial distraction area after 2 (*n* = 6) and 4 (*n* = 6) weeks of consolidation and treatment. The data were confirmed by Student’s *t*-test between control group and ADM2 group. *P < 0.05, **P < 0.01.

The mechanical properties of fresh tibia specimens manifested the stimulative effect of ADM2 on bone consolidation during DO, as evidenced by the improved ultimate load, energy to failure, and E-modulus observed in the ADM2 group (Figure 6A). In addition, X-ray films across the consolidation phase showed bone regeneration progression. At the end of distraction, little callus formation was observed within the distraction area in both groups (Figure 6B). Over time, more mature calluses appeared in the ADM2 group than in the control group in terms of volume and continuity, especially at the end of the consolidation phase (Figure 6B). Similar observations were confirmed using micro-CT examination of the distraction area at 2 and 4 weeks after distraction (Figure 6C). The BMD and BV/TV of distraction regenerates in the ADM2 group were significantly higher than those in the control group after 2 and 4 weeks of consolidation (Figure 6D), suggesting the bone-regenerating effect of ADM2 in the rat DO model.

### Vascularized Bone Regeneration Enhanced by ADM2 Within the Distraction Area

**FIGURE 7:**
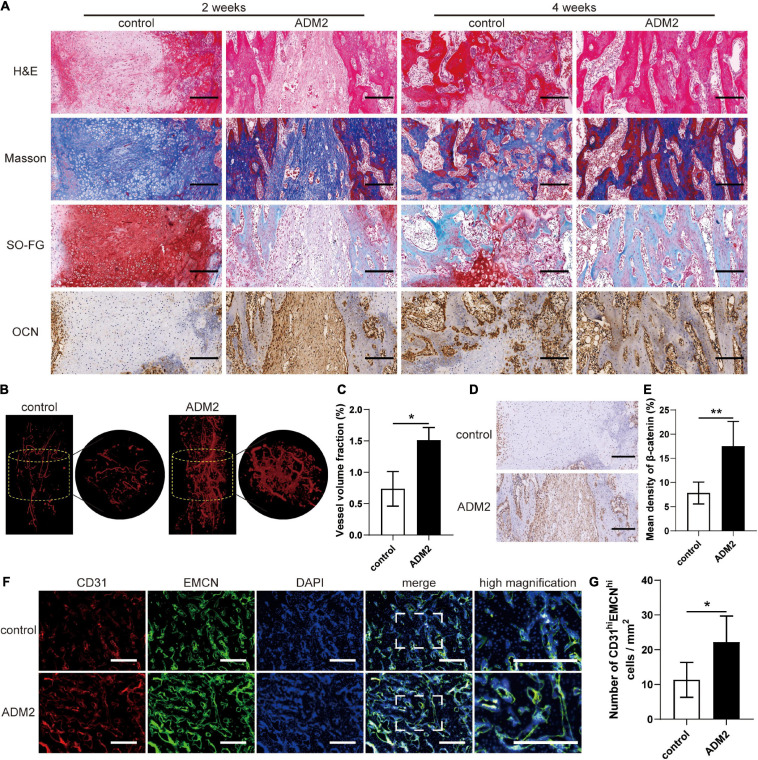
ADM2 improves vascularized bone regeneration within the tibial distraction area. **(A)** Representative images of H&E, Masson, and Safranin O-Fast Green staining and immunohistochemical analysis of OCN in control and ADM2 groups. Scale bar: 200 μm. **(B)** Micro-CT observation of the newly formed blood vessels perfused with Microfil in the distraction regions. **(C)** Quantitative analysis of the vessel volume fractions within the distraction gaps from each group (*n* = 3). **(D)** Immunohistochemical analysis of β-catenin in control and ADM2 groups. **(E)** Quantitative analysis of the immunohistochemical staining of β-catenin (*n* = 3). **(F)** Immunofluorescence staining images of CD31 and EMCN for the distraction area sections from each group. Scale bar: 400 μm. **(G)** Quantitative analysis of CD31^hi^EMCN^hi^ cells per mm^2^ from the staining results (*n* = 3). The data were confirmed by Mann–Whitney *U*-test between control group and ADM2 group. *P < 0.05, **P < 0.01.

H&E, Masson’s trichrome, and SO-FG staining of the distraction regenerates revealed various amounts of newly formed trabecular bone, cartilaginous tissue, and fibrous-like tissue, parallel with the distraction forces (Figure 7A). However, distraction regenerates of ADM2-treated rats exhibited enhanced bone regeneration after 2 and 4 weeks of consolidation, in comparison with the control group, which was evidenced by more neo-formed trabecular bone and less cartilaginous and fibrous-like tissue in the ADM2 group (Figure 7A). Additionally, after 2 weeks of consolidation, a higher expression of OCN, especially around the neo-formed trabecular bone, within the distraction areas of the ADM2 group was confirmed using immunohistochemical analysis, demonstrating the active osteogenic process of ADM2-treated rats in the middle of the consolidation phase (Figure 7A). Thereafter, the expression of OCN in the control group was elevated, indicating that active osteogenesis was processed in the distraction zone of the control group, although 2 weeks later than the ADM2 group. Regarding neovascularization, there were evidently more neo-vessels, which was confirmed by an elevated vessel volume fraction, growing into the distraction areas of the ADM2 group than in the control group (Figures 7B,C). Similarly, CD31 and EMCN immunofluorescence double staining revealed a greater population of CD31^hi^Emcn^hi^ cells within the distraction regenerates of the ADM2 group (Figures 7D,E). Meanwhile, the number of type-H endothelial cells in the metaphysis of the ADM2 group was also significantly higher than that in the control group (Supplementary Figure 3), corroborating the enhancive effect of ADM2 on the coupling of osteogenesis and angiogenesis. Additionally, immunohistochemical analysis revealed a higher expression of β-catenin within the distraction regenerates of the ADM2 group (Figures 7F,G) in the middle of the consolidation phase, suggesting that β-catenin signaling contributed to the enhancement in the coupling of osteogenesis and angiogenesis induced by ADM2 treatment during DO.

## Discussion

In the present study, we found that ADM2 enhanced the osteogenic differentiation and the pro-angiogenic potential of BMSCs. In addition, the effects of ADM2 on BMSCs were achieved, at least partially, by the accumulation and activation of β-catenin, which was attributed to the inhibition of NF-κB and the activation of AKT. Moreover, the *in vivo* improvement effects of ADM2 on bone regeneration and vessel expansion in DO rats were also verified. To the best of our knowledge, this is the first study to provide evidence that ADM2 enhances the BMSC-mediated coupling of osteogenesis and angiogenesis through β-catenin signaling.

**FIGURE 8:**
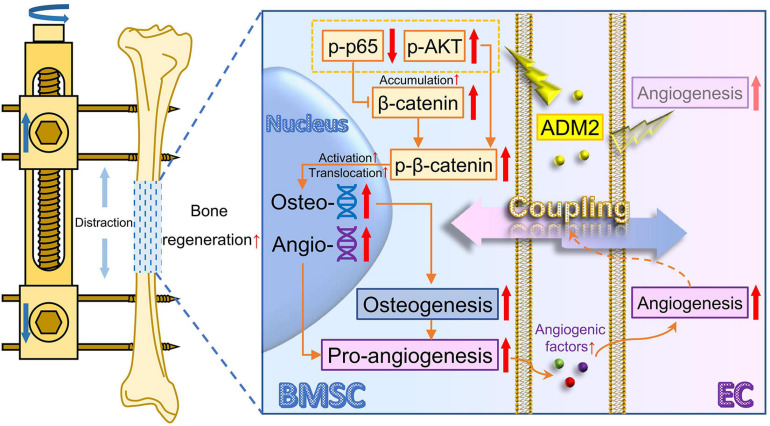
Working model of ADM2 promoting the coupling of osteogenesis and angiogenesis during DO through NF-κB/β-catenin and AKT/β-catenin signaling. Under the administration of ADM2, NF-κB signaling is inhibited and AKT signaling is activated, leading to the accumulation and activation of β-catenin in BMSCs. Therefore, the osteogenic differentiation and pro-angiogenic potential of BMSCs are promoted, and the tube formation and cell motility of ECs are enhanced. In conclusion, ADM2 could directly promote osteogenesis and indirectly improve angiogenesis through β-catenin signaling, thus enhancing BMSCs-mediated coupling of osteogenesis and angiogenesis and accelerating bone regeneration during DO. ADM2, adrenomedullin 2. AKT, activate protein kinase B. BMSC, bone marrow mesenchymal stem cell. DO, distraction osteogenesis. EC, endothelial cell. NF-κB, nuclear factor kappa-light-chain-enhancer of activated B cells.

Considerable evidence has shown that BMSCs play a vital role in bone regeneration in terms of osteogenesis (Zhang L. et al., 2017; Iaquinta et al., 2019). As the knowledge about the coupling of osteogenesis and angiogenesis is expanding, osteoblasts, differentiated from BMSCs, have been identified as important regulatory cells to enhance angiogenesis and the expansion of type-H vessels (Xu et al., 2018; Li et al., 2020), which also contribute to a satisfactory regeneration of bone (Wang et al., 2017). In other words, except for being directly involved in mineralization, BMSCs could improve the substance supply and signaling regulation within the regenerative microenvironment through enhanced pro-angiogenic ability upon osteogenic differentiation, indirectly enhancing osteogenesis. Therefore, the present study generated simultaneous improvement for osteogenic differentiation and pro-angiogenic potential of BMSCs, which intervenes the direct and indirect factors participating in osteogenesis, as the therapeutic strategy, thereafter verified that ADM2 could indeed promote bone regeneration during DO by exerting this dual stimulative effect through β-catenin signaling (Figure 8).

As a vital transcription factor in the wingless/integrated (Wnt) pathway, β-catenin plays an important role in osteogenesis and angiogenesis (Houschyar et al., 2018), and its activation state can be regulated by a variety of upstream signals (Clevers and Nusse, 2012; Xue et al., 2015; Huang et al., 2019). Wang et al. (2019) found that Wnt/β-catenin signaling plays an important role in ADM2-induced neovascularization of endothelial cells, while the mechanisms by which ADM2 mediates β-catenin remain poorly understood. Previous studies have verified that NF-κB signaling could promote β-catenin ubiquitination and degradation, while AKT signaling could activate β-catenin (Chang et al., 2013; Zheng et al., 2018). Of note, ADM2 has been widely reported to mediate the inhibition of NF-κB and the activation of AKT (Song et al., 2009; Li et al., 2013); therefore, we speculated that ADM2 possesses the potential to activate the Wnt pathway by simultaneously modulating both upstream signals. Indeed, in the present study, ADM2 treatment significantly inhibited NF-κB and activated AKT in BMSCs upon osteogenic induction, leading to an enhanced accumulation and activation of β-catenin, along with an improved osteogenic differentiation and pro-angiogenic potential. As expected, the effects of ADM2 could be attenuated by the TNF-α-mediated activation of NF-κB and the MK2206-mediated inhibition of AKT. In the DO model, ADM2 administration distinctly improved bone regeneration and vessel expansion within distraction regenerates, accompanied by β-catenin activation, indicating that ADM2 enhances the coupling of osteogenesis and angiogenesis *in vivo*. Notably, previous studies have verified that AKT could regulate the activation of NF-κB as an upstream signaling molecule (Tang et al., 2016; Balaji et al., 2018; Ji et al., 2019). The present study demonstrated that MK2206 attenuated the activation of AKT induced by ADM2, and further inhibited NF-κB signaling, although not resulting in a significant change in the expression of β-catenin. However, ADM2 indeed activated AKT while inhibiting NF-κB signaling simultaneously in the present study, indicating that ADM2-mediated variations in NF-κB and AKT signaling were relatively correlated but primarily independent, although the detailed mechanisms have not been clarified. Moreover, β-catenin signaling could be regulated by various pathways, in addition to NF-κB and AKT (Ghahhari and Babashah, 2015; Xue et al., 2015; Fu et al., 2018). Therefore, the comprehensive mechanism underlying the accumulation and activation of β-catenin induced by ADM2 remains to be explored.

Osteoblasts play an important role in promoting angiogenesis in the skeletal system based on the theoretical system of the coupling between osteogenesis and angiogenesis (Xu et al., 2018). However, BMSCs, which are the precursors of osteoblasts, have also been generally employed to promote angiogenesis in previous studies (Sharma et al., 2018; Zhang et al., 2018). The variation in the pro-angiogenic potential of BMSCs during osteogenic differentiation is disputable, leading to a dubious mechanism by which ADM2 improves the pro-angiogenic potential of BMSCs upon osteogenic differentiation. The enhanced pro-angiogenic ability of the CM from BMSCs may be attributed to the improved pro-angiogenic potential of osteoblastic lineage cells or to the increased population of mature osteoblasts. As evident from our results, only the expression of *VEGFA*, among multiple angiogenic genes, was downregulated in BMSCs during osteogenic differentiation. However, ADM2 treatment not only promoted the osteogenic differentiation of BMSCs, but also rescued the gene expression of *VEGFA*, which was supposed to be further downregulated due to an enhanced osteogenic differentiation, suggesting that the pro-angiogenic potential of BMSCs was gradually stimulated by OIM induction and ADM2 treatment via β-catenin signaling. Meanwhile, the enhanced pro-angiogenic potential of BMSCs induced by ADM2 treatment alone also corroborates this conclusion. However, the pro-angiogenic ability of BMSCs is associated with multiple mechanisms, including the exocrine and paracrine mechanisms of a variety of cytokines (Chen et al., 2015; Sharma et al., 2018) and the release of exosomes (Zhu et al., 2019; Yu et al., 2020). Here, we only selected several representative angiogenic factors for testing, although the ability and mechanism of BMSCs to promote angiogenesis, upon osteogenic differentiation and ADM2 treatment, still need to be further verified using high-throughput sequencing technology.

The activities of the calcitonin family peptides in bone are mainly associated with osteoclastogenesis, which could be inhibited by calcitonin, CGRP, amylin, and ADM2, through cyclic adenosine monophosphate (cAMP) signaling (Naot et al., 2019). In addition, the pharmacological effects of the calcitonin family peptides on osteogenesis and angiogenesis have also been widely investigated. For instance, ADM2 was reported to directly improve the angiogenic activity of endothelial cells (Wang et al., 2018), and CGRP, which shares CLR/RAMP1 as a common receptor with ADM2 (Naot et al., 2019), could improve osteogenesis and angiogenesis through the Wnt (Zhou et al., 2016) and AKT (Mi et al., 2021) pathways, respectively. Meanwhile, ADM also possesses similar angiogenesis-promoting functions as ADM2 (Zhang Y. et al., 2017), although the additional activities of ADM on improving osteogenesis (Cornish et al., 2001) may not be mediated by its common receptors with ADM2, since CLR/RAMP1 is not one of the receptors ADM prefers to combine with and there is no expression of CLR/RAMP3 in BMSCs and osteoblasts (Naot and Cornish, 2008). Since, in the present study, we verified the stimulatory effects of ADM2 on osteogenic differentiation and the pro-angiogenic potential of BMSCs, and the reported intrinsic activities of ADM2 on osteoclastogenesis and angiogenesis are potentially associated with type-H endothelial cells (Xie et al., 2014), the physiological role of ADM2 in the coupling of osteogenesis and angiogenesis has been gradually established. Based on this, due to the common receptors and similar biological activities of most peptides of the calcitonin family (Naot et al., 2019), this study, in which the role of ADM2 in the coupling of osteogenesis and angiogenesis was investigated, is expected to link the calcitonin family peptides with type-H endothelial cells. This means that various physiological functions of type-H endothelial cells can be adopted as potential pharmacological activities of calcitonin family peptides, targeting bone defects, osteoporosis, and even bone metastasis (Singh et al., 2019). Moreover, the present exploration of the AKT- and NF-κB-mediated accumulation and activation of β-catenin not only supplements the mechanisms by which ADM2 and other calcitonin family peptides may potentially regulate osteogenesis and angiogenesis, but also provide novel insights into the relationship between calcitonin family peptides and osteoclastogenesis, except for the cAMP pathway, according to the inhibitory effect of β-catenin signaling on osteoclastogenesis (Yan et al., 2020).

There are several limitations in the present study. First, the detailed mechanism underlying the ability of ADM2 to inhibit NF-κB and activate AKT remains to be fully elucidated. Second, the pro-angiogenesis-promoting function of ADM2 in osteoblasts has not been separately investigated. Third, as an endogenous peptide, the physiological role and variation of ADM2 in the process of bone regeneration during DO, for optimizing the administration doses and the treatment timing, have not been clarified. Consequently, further studies should target the physiological role and molecular mechanism underlying the therapeutic effects of ADM2 to further understand the role of ADM2 to accelerate the translation of ADM2-related biological therapies into clinical settings.

## Conclusion

The present study demonstrates that ADM2 improves the osteogenic differentiation and pro-angiogenic potential of BMSCs through the accumulation and activation of β-catenin by inhibiting NF-κB and activating AKT signaling, revealing that ADM2 is a novel bioactive factor that shortens the consolidation phase during DO treatment. Moreover, our study aims to provide a novel therapeutic strategy for accelerating bone regeneration by improving the coupling of osteogenesis and angiogenesis through β-catenin signaling in BMSCs.

## Data Availability Statement

The raw data supporting the conclusions of this article will be made available by the authors, without undue reservation.

## Ethics Statement

The animal study was reviewed and approved by Animal Research Committee of Shanghai Jiao Tong University Affiliated Sixth People’s Hospital.

## Author Contributions

QK and JX conceived and designed the experiments. FW and WW performed the experiments. QK, JX, FW, and WW wrote the manuscript. FW, WW, JX, and QK analyzed the data and prepared all the figures. LK, LS, and MW provided the technical support. YC provided the financial support. All the authors reviewed and agreed upon the manuscript.

## Conflict of Interest

The authors declare that the research was conducted in the absence of any commercial or financial relationships that could be construed as a potential conflict of interest.

## Supplementary Material

Supplementary Figure 1 Characterization of the cell population of BMSCs. **(A)** BMSCs were induced to differentiate to three cell lineages and stained with alizarin red for osteogenesis, alcian blue for chondrogenesis, and oil red O for adipogenesis to verify the multiple differentiation potential. Scale bar: 100 μm. **(B)** The expression of specific surface markers of BMSCs was examined using flow cytometry.

Supplementary Figure 2 Gene expression of *CLR*, *RAMP1*, and *RAMP3* in BMSCs treated with OIM and different concentrations of ADM2 was assessed using qRT-PCR. The data were confirmed by one-way analysis of variance (ANOVA) followed by Tukey’s *post hoc* test from three independently repeated tests and are presented as the means ± SD. ^NS^*P* > 0.05, **P* < 0.05, ***P* < 0.01.

Supplementary Figure 3 ADM2 improves the number of type-H endothelial cells within the metaphyseal region. **(A)** Immunofluorescence staining images of CD31 and EMCN for the metaphyseal region sections from each group. Scale bar: 100 μm. **(B)** Quantitative analysis of CD31^hi^EMCN^hi^ cells per mm^2^ from the staining results (*n* = 3). The data were confirmed by Mann–Whitney *U*-test between control group and ADM2 group. ^∗∗^*P* < 0.01. BM, bone marrow. GP, growth plate.

## Abbreviations

ADM2: adrenomedullin 2
AKT: activate protein kinase B
ALP: alkaline phosphatase
Ang: angiopoietin
ARS: alizarin red S
BMD: bone mineral density
BMCS: bone marrow mesenchymal stem cell
BSA: bovine serum albumin
BV/TV: bone volume/tissue volume
cAMP: cyclic adenosine monophosphate
CD31: platelet endothelial cell adhesion molecule-1
CGRP: calcitonin gene related peptide
CM: conditioned medium
CLR: calcitonin receptor-like receptor
CT: computed tomography
DAPI: 4’,6-diamidino-2-phenylindole
DO: distraction osteogenesis
EDTA: ethylene diamine tetraacetic acid
ELISA: enzyme-linked immunosorbent assay
EMCN: endomucin
E-modulus: modulus of elasticity
FBS: fetal bovine serum
GAPDH: glyceraldehyde-3-phosphate dehydrogenase
GSK-3 β: glycogen synthase kinase-3 β
H&E: hematoxylin-eosin
HRP: horseradish peroxidase
NF- κ B: nuclear factor kappa-light-chain-enhancer of activated B cells
OCN: osteocalcin
OIM: osteogenic induction medium
OPN: osteopontin
OSX: osterix
P/S: penicillin-streptomycin
PBS: phosphate-buffered saline
PFA: paraformaldehyde
qRT-PCR: quantitative real-time polymerase chain reaction
RAMP: receptor-modifying protein
RUNX2: runt-related transcription factor 2
SD: Sprague-Dawley
SDF-1: stromal cell derived factor-1
Slit3: slit homolog 3
Smurf: smad ubiquitin regulatory factor
SO-FG: safranine O-fast green
TNF- α: tumor necrosis factor-alpha
VEGFA: vascular endothelial growth factor A.
