# Supplementary figures and images for: Accelerated Bone Regeneration by Adrenomedullin 2 Through Improving the Coupling of Osteogenesis and Angiogenesis via β-Catenin Signaling
Source: Front Cell Dev Biol. 2021 Apr 14;9:649277. doi: 10.3389/fcell.2021.649277 (PMC8079771; doi:10.3389/fcell.2021.649277)

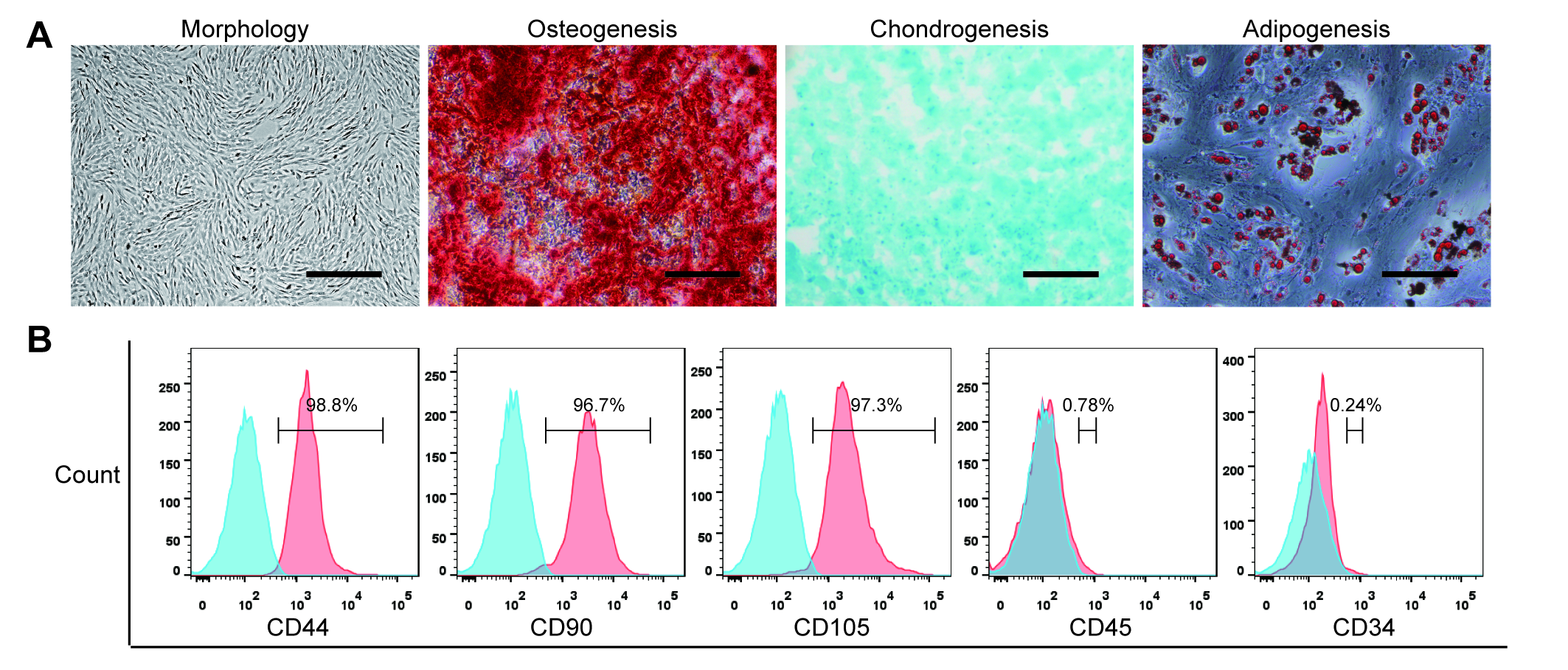

Supplement: Supplementary Figure 1 — Characterization of the cell population of BMSCs. (A) BMSCs were induced to differentiate to three cell lineages and stained with alizarin red for osteogenesis, alcian blue for chondrogenesis, and oil red O for adipogenesis to verify the multiple differentiation potential. Scale bar: 100 μm. (B) The expression of specific surface markers of BMSCs was examined using flow cytometry. [file Image_1.TIF]

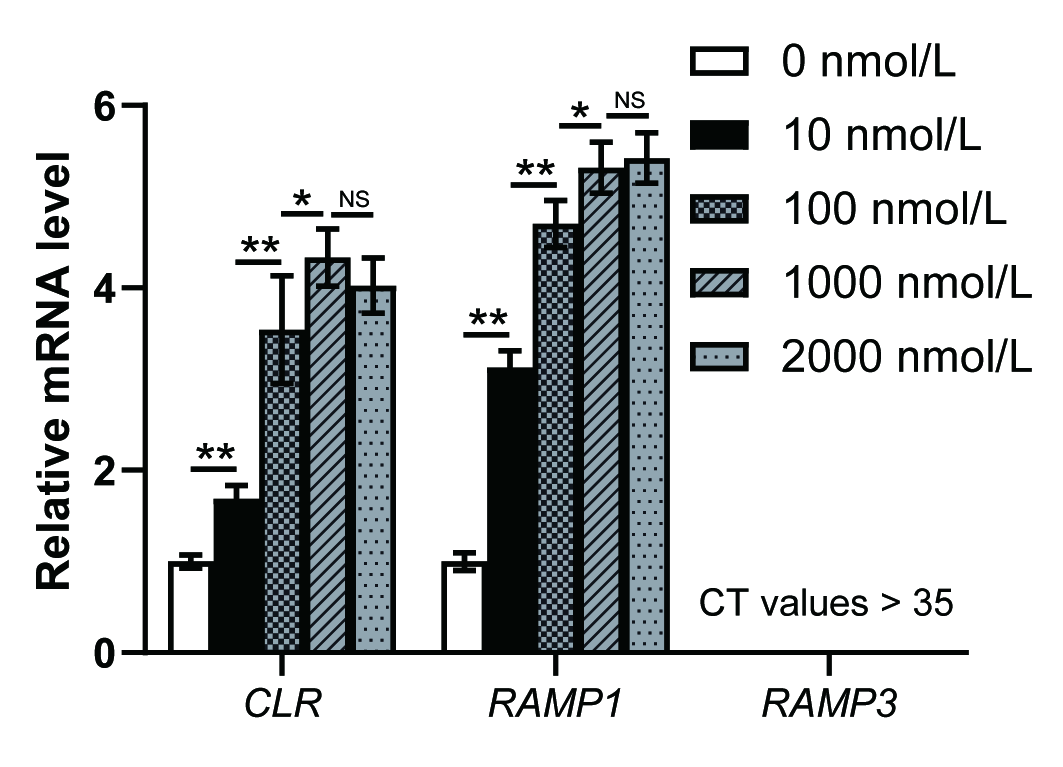

Supplement: Supplementary Figure 2 — Gene expression of CLR, RAMP1, and RAMP3 in BMSCs treated with OIM and different concentrations of ADM2 was assessed using qRT-PCR. The data were confirmed by one-way analysis of variance (ANOVA) followed by Tukey’s post hoc test from three independently repeated tests and are presented as the means ± SD. NSP > 0.05, *P < 0.05, **P < 0.01. [file Image_2.TIF]

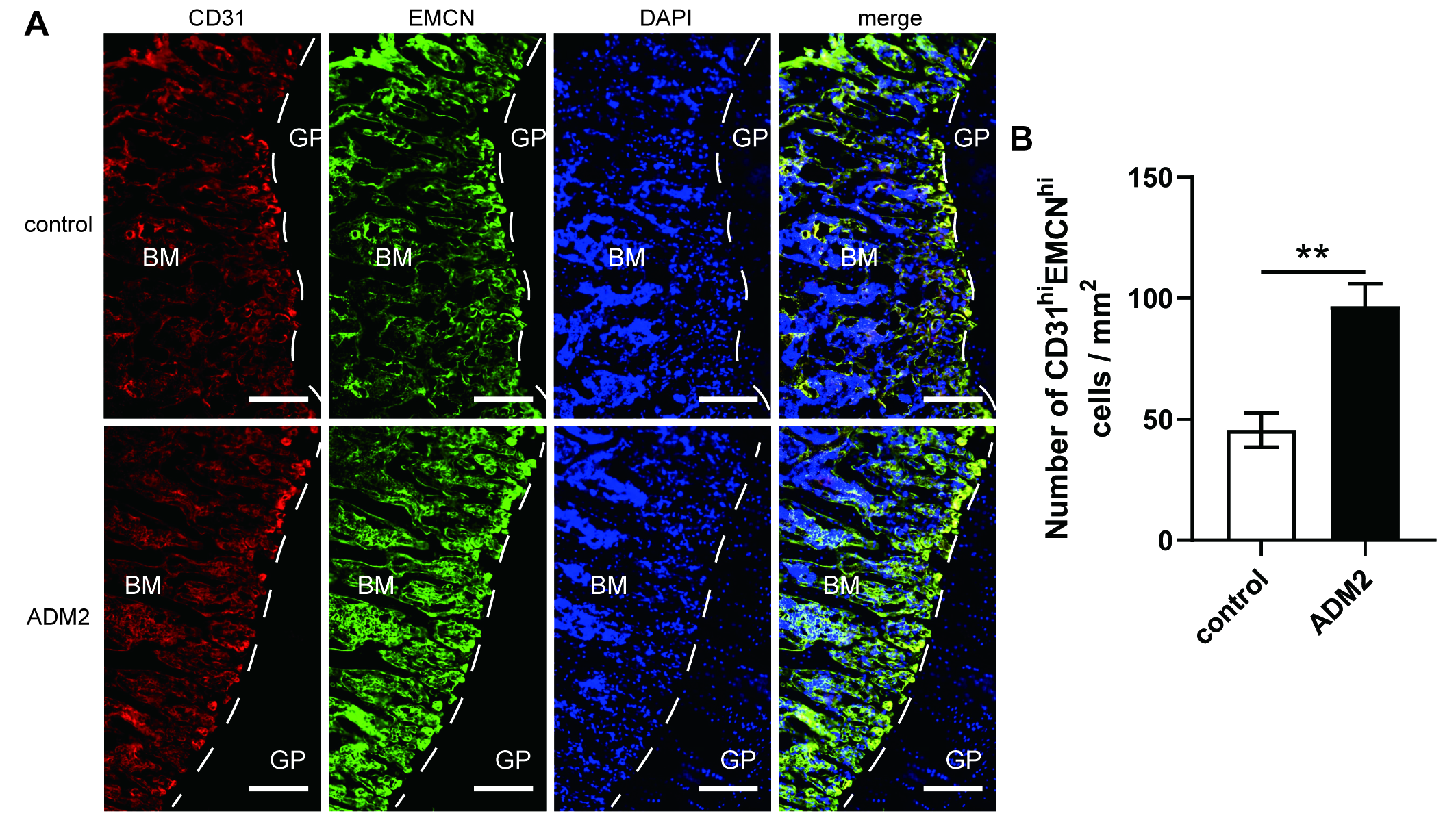

Supplement: Supplementary Figure 3 — ADM2 improves the number of type-H endothelial cells within the metaphyseal region. (A) Immunofluorescence staining images of CD31 and EMCN for the metaphyseal region sections from each group. Scale bar: 100 μm. (B) Quantitative analysis of CD31hiEMCNhi cells per mm2 from the staining results (n = 3). The data were confirmed by Mann–Whitney U-test between control group and ADM2 group. ∗∗P < 0.01. BM, bone marrow. GP, growth plate. [file Image_3.TIF]
